# Pathogen-driven gastrointestinal cancers: Time for a change in treatment paradigm?

**DOI:** 10.1186/1750-9378-7-18

**Published:** 2012-08-08

**Authors:** Bauyrzhan Aituov, Assem Duisembekova, Assel Bulenova, Kenneth Alibek

**Affiliations:** 1 Nazarbayev University, 53 Kabanbay Batyr Avenue, Astana 010000, Kazakhstan; 2 Republican Scientific Center for Emergency Care, 3 Kerey and Zhanibek Khan Street, Astana 010000, Kazakhstan

**Keywords:** Gastric cancer, Colorectal cancer, Liver cancer, Helicobacter pylori, Cytomegalovirus, Epstein-Barr virus, John Cunningham virus, *Streptococcus bovis*, Hepatitis C virus, Helminthes

## Abstract

The regulation of cancerous tumor development is converged upon by multiple pathways and factors. Besides environmental factors, gastrointestinal (GI) tract cancer can be caused by chronic inflammation, which is generally induced by bacteria, viruses, and parasites. The role of these inducers in cancer development, cell differentiation and transformation, cell cycle deregulation, and in the expression of tumor-associated genes cannot be ignored. Although *Helicobacter pylori* activates many oncogenic pathways, particularly those in gastric and colorectal cancers, the role of viruses in tumor development is also significant. Viruses possess significant oncogenic potential to interfere with normal cell cycle control and genome stability, stimulating the growth of deregulated cells. An increasing amount of recent data also implies the association of GI cancers with bacterial colonization and viruses. This review focuses on host-cell interactions that facilitate primary mechanisms of tumorigenesis and provides new insights into novel GI cancer treatments.

## Review

### A. Gastric Cancers

Despite many groundbreaking discoveries in biomedical research, cancer is still a major health risk. Gastrointestinal (GI) cancers account for more than 20% of all cancers worldwide [1], and this holds true for both developing and developed countries [2].

Although genetic predisposition and lifestyle are considered the leading risk factors for cancer development, increasing clinical data suggest that pathogens may play a more significant role than previously thought. In this review, we focus on the implication of pathogens in tumor development in the three major GI cancers–gastric, colorectal, and liver.

#### A1. Gastric cancer (GC) and bacterial infections

*Helicobacter pylori* colonization of the human gastric mucosa occurs in 50% of the human population and is a key factor in GC development. Having been categorized as a class I carcinogen by the World Health Organization in 1994, and this categorization has been reiterated by the International Agency for Research on Cancer in 2010 [3], *H. pylori* possess virulence factors such as the cytotoxin-associated gene (cagA) and vacuolating cytotoxin gene (vacA) [4]. The oncoprotein CagA and the type IV secretion machinery are encoded by the cag pathogenicity island (cag PAI). *H. pylori* employs type IV secretion machinery to insert CagA into the host cell cytoplasm, thus causing cell proliferation, morphologic alterations, and cell motility [5]. These processes are further linked to morphologic alterations of the host cell, such as the loss of cell polarity, dissolution of cellular junctions, remodeling of the extracellular matrix, and activation of the β-catenin pathway, thus conferring an oncogenic potential to the cell [6]. Parsonnet *et al. *[7] and Torres *et al. *[8] reported that CagA-seropositive GC patients have a higher risk of cancer compared to CagA-seronegative patients. *H. pylori* also transmits Vac A, a bacterial toxin, which inhibits glycogen synthase kinase 3-β-regulated signaling leading to β-catenin release and altered apoptosis as well as cell-cycle regulation [9,10]. Additionally, *H. pylori* expresses an outer-membrane protein, BabA, which may cause enhanced inflammation and dense bacterial colonization [11]. Therefore, *H. pylori* strains that possess CagA, VacA, and BabA proteins confer a greater risk of GC induction.

CagA can also interact with VacA to cause the deregulation of nuclear factor of activated T-cell signaling [12]. This leads to p21 expression, which influences the fate of cell cycle and cell differentiation. CagA binds to partitioning-defective and mitogen-activated protein/microtubule affinity-regulating kinase complexes, resulting in reduced kinase activity and disturbed cell polarity [13]. CagA interaction with E-cadherin affects β-catenin signaling, leading to intestinal trans-differentiation [14].

Ooi *et al. *[15] reported the activation of three main oncogenic pathways in the majority of GC patients: (1) proliferation/stem cells were activated in 40% of the patients, (2) nuclear factor kappa beta (NF-κβ) activity was stimulated in 39% of the patients, and (3) Wnt/β-catenin activity was stimulated in 46% of the GC patients. Deregulation of these three pathways was observed in more than 70% of the patients diagnosed with GC, which resulted in increased inflammatory cytokine production, abnormal apoptosis, undesirable epithelial cell proliferation/differentiation, and epithelial cell transformation [15].

*H. pylori* infection causes the activation of oncogenic pathways, thus leading to the aberrant expression of genes that are crucial in gastric carcinogenesis. Hirata *et al. *[16] found that NF-kβ is activated by *H. pylori* and is observed in the majority of GCs. The relationship between *H. pylori*-induced inflammation and oncogenic mechanisms of the Wnt/β-catenin and prostaglandin E2 pathways has been established [17]. It is implicated that the activation of both the Wnt and prostaglandin E2 pathways results in the formation of gastric tumors via the metaplasia-carcinoma sequence. CagA deregulates the Wnt/β-catenin pathway, and upon interaction with E-cadherin, it destroys the formation of the complex between E-cadherin and β-catenin. This further causes the activation of the cdx1 and p21 genes and promotes the aberrant expression of goblet-cell mucin (MUC2) [14], an intestinal differentiation marker.

*H. pylori* interferes with epigenetic regulation, particularly via microRNAs (miRNAs). miRNAs are small, non-coding RNA molecules that are involved in the post-transcriptional regulation of gene expression during the processes of cell proliferation and development. Specific miRNAs are recognized as tumor suppressors, because their expression is altered in cancer phenotypes [18]. *H. pylori* has evolved a mechanism to hijack miRNA, thus suppressing host cellular functions to establish infection. It has recently been observed that miR-21 was upregulated in cells obtained from GC patients and in tissues from patients who were chronically infected with *H. pylori *[18]. miR-21 targets the phosphatase and tensin homolog phosphatase and actin-binding protein tropomyosin I, which are tumor suppressors. Consequently, miR-21 aids in the survival of deregulated cells. It has been reported for the first time that miR-21 is altered in *H. pylori* infection [18]. Ectopic over-expression of miR-21 promoted cell proliferation and inhibited apoptosis.

According to a study by Li *et al. *[19], another type of miRNA, miR-222, is upregulated in *H. pylori*-infected GC. miR-222 is known to participate in the progression of cancer by promoting cell proliferation [19], which suggests that *H. pylori* may serve as a cancer inducer by up regulating miR-222. Till date, only miR-21 and miR222 are the two types of miRNAs known to be upregulated in *H. pylori* infection. Further studies are required to investigate the exact role of microRNAs in gastric carcinogenesis.

In addition, there is growing evidence that *Helicobacter spp* plays a significant role in the bacterial cause of GC in rodents [20]. *H. felis* is known to cause GC in C57BL/6 mice, which exhibit a histological progression of cancer similar to that observed in *H. pylori*-infected human subjects. *H. felis* is different from *H. pylori* in that *H. felis* does not possess PAI and *cag* genes.

#### A2. GC and viral infections

The presence of Epstein-Barr virus (EBV) in the neoplastic cells in GC is defined as EBV-associated gastric carcinoma (EBVaGC). It is estimated that 10% of the total GC cases are related to EBVaGC, and more than 90,000 patients are diagnosed annually with EBVaGC [21]. There is a strong association between the presence of EBV and gastric carcinoma due to the oncogenic properties of the virus [22]. EBV encodes for the latent viral products Epstein-Barr nuclear antigen 1, Epstein-Barr virus-encoded small RNAs (EBERs), and latent membrane protein 2A (LMP2A), as well as encodes for transcripts from the BamH1 A region, such as EBV-encoded BamH1-A reading frame-1.

Epstein-Barr nuclear antigen 1 is constitutively expressed in EBVaGC, because it is vital for maintaining EBV replication in the host [23]. EBERs are present in all patients diagnosed with EBVaGC [24-26]. EBER1 upregulated the expression of insulin-like growth factor, thus promoting the growth of NU-GC-3 gastric cancer cells [27].

LMP2A was reported to be expressed in 50% of all EBVaGC cases [28,29]. It upregulated DNA methyl transferase 1 through signal transducer and activator of transcription 3 phosphorylation, which causes promoter hypermethylation of phosphatase and tensin homolog, a tumor suppressor gene [30]. In addition, following EBV infection, LMP2A increases cell survival in GC cell lines, thus making them resistant to serum deprivation-induced apoptosis [31]. LMP2A plays an important role in carcinogenesis; it modifies normal B-cell development and maintains EBV latency [32]. The regulation of viral and cellular gene expression through altered NF-κB activity implies an essential role of LMP2A in carcinogenesis [33]. EBV-encoded BamH1-A reading frame-1 leads to an increase in the bcl-2/bax ratio, and as a result, GC cells escape apoptosis [34]. EBVaGCs are resistant to apoptosis compared to EBV-negative GCs, which suggests that resistance to apoptosis is an essential feature of EBVaGCs [35,36]. Moreover, a higher methylation rate of tumor-related genes such as p14ARF, p15, p16INK4A, TIMP3, E-cad, DAPK, and GSTP1 was observed in EBVaGCs compared to EBV-negative GCs [31]. In terms of clinopathologic characteristics, EBVaGC is predominant in males, and the most common location is the proximal stomach, with a high frequency found in diffuse-type gastric adenocarcinoma. Chen *et al. *[37] showed that the frequency of EBVaGC is significantly higher in gastric remnant carcinoma (GRC) than in conventional gastric carcinoma.

Another virus that is reported to play a role in GC is the human polyomavirus called the John Cunningham virus (JCV). JCV induces oncogenesis by expressing the “transforming antigen” (T-Ag). T-Ag acts as a carcinogen by interacting with p53 and the cell-cycle regulator pRb, which consequently leads to an uncontrolled growth of cancerous cells. This leads to the loss of genomic stability, which is manifested as an activation of oncogenes and inactivation of tumor suppressor genes. JCV T-Ag interferes with the genome stability of the cell through the inhibition of homologous recombination during DNA repair [38]. Furthermore, it has been demonstrated that more than 50% of GC patients show chromosomal instability including loss of heterozygosity and various chromosomal rearrangements [39,40]. Shin *et al. *[41] revealed the presence of JCV T-Ag in gastric tissues, where T-Ag was present in 21 out of 37 GC patients (57%). This suggests an association between JCV polyomavirus and GCs. Other studies have shown that JCV T-Ag DNA sequences are present in 80% to 90% of colorectal cancers (CRCs) [42,43].

#### A3. GC and parasitic infections

The human digestive system is densely populated not only with bacteria and viruses, but also with parasites. The role of parasites in GC tumor progression has been recently elucidated. Toxocariasis was confirmed serologically in five male patients, of which three had GC, and two had CRC [44]. Additionally, there was a case report where *Microfilaria* was found in a 55-year-old man diagnosed with gastric carcinoma, although the underlying mechanism of tumor development still needs to be investigated [45]. *Tropheryma whippelii* has been strongly associated with specific cases of gastric adenocarcinoma; however, the frequency of this association is considered to be low [46].

### B. Colorectal cancer

According to the American Cancer Society, CRC is the fourth most common cancer in men and the third most common cancer in women, worldwide [20]. Epidemiological data accumulated so far has established an association between CRC development and various environmental factors, such as high-calorie diet and obesity; however, the data seems to be contradictory, and these are not recognized as high-risk factors [47]. Progression to CRC, similar to other cancers, is a multistep process, often with a background of genomic instability. Several molecular hallmarks are characteristic of sporadic CRCs that include chromosomal and microsatellite instability, together with epigenetic silencing via CpG methylation [48]. In contrast to other GI cancers, a direct causal association between CRC development and pathogen infection (bacteria and viruses) has not yet been established [49]. Therefore, extensive studies have been carried out in the past decade to gain more insight into this field.

#### B1. CRC and bacterial infections

*Streptococcus bovis* bacteremia was associated with both colonic neoplasia and extra colonic malignant diseases, despite the fact that it is known as a commensal of the human GI tract [50]. Antibodies to *S. bovis* surface antigens have been identified in patients with CRC, where increased IgG titers were more consistent with a chronic rather than an acute infection [51]. Moreover, *S. bovis* antigens were also detected in polyps, supporting the finding that *S. bovis* infection occurs early during CRC carcinogenesis [51]. As previously mentioned, *H. pylori* is a causal factor in GC development, and therefore might play a role in the pathogenesis of other GI cancers. A number of studies have identified *H. pylori* DNA in biopsies of CRC patients [52,53].

At least three bacterial toxins have been characterized that might trigger cellular proliferation. The *Bacteroides fragilis* toxin disrupts cell cytoskeleton and activates c-Myc and cyclin-D, leading to increased proliferation [54,55]. Cytotoxic necrotizing factor produced by *Escherichia coli* strains activates Rho GTPases and modifies the cytoskeleton, resulting in the stimulation of metastatic activity [56,57]. This toxin also triggers G1-to-S phase transition to induce host genome replication [58]. *H. pylori*-encoded cag PAI toxin and vacuolating cytotoxin were shown to modulate cell division and apoptosis by altering the mitogen-activated protein kinase and epidermal growth factor receptor pathways [59].

Several hypotheses have been proposed to explain the oncogenic potential of bacteria. Inflammation in response to bacterial infection results in elevated production of cytokines and reactive oxygen species (ROS), which in turn leads to the up regulation of cyclooxygenase 2 and NF-κB [60]. ROS production is highly likely to induce neoplastic transformation via DNA damage [61]. Bacteria might either damage DNA or modulate DNA-repair pathways, increasing their susceptibility to somatic mutations [56]. Activated cyclooxygenase 2 and NF-κB generally inhibit apoptosis [62]. Furthermore, ROS generation by commensal bacteria, such as *S. bovis,* might also lead to genomic instability and result in mutations [60].

#### B2. CRC and viral infections

A number of viruses have also been implicated in CRC pathogenesis. These include JCV, EBV, cytomegalovirus (CMV), and human papillomavirus (HPV), and the functional roles of their genes have been identified in various stages of CRC [49]. For instance, it has been previously reported that JCV DNA sequences are frequently present in the upper and lower GI tracts and in CRCs [63]. Recently, it was demonstrated that T-Ag is involved in Wnt signaling by forming a complex with β-catenin, which results in the transcriptional activation of c-Myc and cyclin D1 [64]. It is currently established that the c-myc gene is over-expressed in nearly 70% of CRCs [65]. Furthermore, Goel *et al.* (2006) demonstrated a strong association between T-Ag expression and chromosome instability in CRCs [66]. They also revealed a direct link between T-Ag expression and aberrant DNA methylation in CRCs. These data seem to be consistent with the aforementioned tumor-inducing mechanisms employed by JCV in GC patients.

EBV DNA was identified in 32% of colorectal adenocarcinomas [67], CMV DNA was detected in 80% of carcinomas and polyps [68], and HPV DNA was identified in 60% of adenomas and 97% of carcinomas [69]. These findings were associated with caspase 3 inhibition (in the case of EBV) [67], up regulation of Fos, Jun, and Myc oncogenes (in the case of CMV) [68,70], and inactivation of the tumor suppressors p53 and pRb (in the case of HPV) [71].

### C. Liver cancer

Liver cancer (the most common form known as hepatocellular carcinoma [HCC]) is the sixth most prevalent malignant tumor in the world, with more than 600,000 new cases being diagnosed each year, and is the third most common cause of cancer-related mortality [72]. HCC is a malignant cancer with a poor prognosis and develops as a complication of liver cirrhosis [73]. Multiple mechanisms have been reported to contribute to the process of liver carcinogenesis [74].

When the regulation of cell growth is impaired, uncontrolled cell division results in HCC. Mutations in growth factors, together with chronic cell injury and regeneration, cause excessive hepatocyte proliferation [75]. Subsequently, immortal cells emerge that are susceptible to DNA damage by ROS or environmental factors, eventually leading to malignant hepatocyte transformation [75]. Although some pathophysiological aspects of liver carcinogenesis are known, we still lack a comprehensive picture of the process.

#### C1. HCC and viral infections

Although alcohol-related diseases are known to cause HCC, infection with hepatitis C and hepatitis B viruses (HCV and HBV) is the most common risk factor [76]. Despite a significant decline in the rates of HBV infection and alcohol-related diseases, HCV alone still accounts for most HCC cases in the developed world [77,78]. In addition to causing HCC, HCV infection is shown to induce severe liver fibrosis and cirrhosis, as well as GI bleeding [79]. As in GC and CRC, various mechanisms are implicated in the host-viral interactions that lead to HCC progression [80].

Experimental data suggests that HCV causes malignant transformation of hepatocytes directly by regulating different signaling pathways [81]. For instance, HCV-encoded proteins, such as non-structural proteins 3, 4B, and 5A, were shown to modulate oncogenic pathways leading to hepatocellular transformation [81].

In HCC patients, the HCV core and NS5A proteins have been reported to induce accumulation of β-catenin molecules that results in impaired Wnt-β-catenin signaling [81]. As mentioned previously, a similar strategy is employed by viral and bacterial pathogens in GC and CRCs. Furthermore, the HCV core protein was reported to interact directly with p53, p21, and NF-κB [82]. As mentioned previously, pathogens adapt similar mechanisms to modulate signaling pathways in GC and CRCs.

#### C2. HCC and fungal infections

Environmental toxins such as fungal aflatoxin are shown to be carcinogenic in some parts of Asia and Africa [77,83]. Recently, the G249T mutation in the p53 gene was found in more than 50% of HCC patients from Southern Africa and some parts of China [84,85]. This mutation results in the expression of a defective p53 and is linked with aflatoxin B1 (AFB1) contamination in the local food [86]. In other geographic regions with undetectable or low levels of AFB1 mycotoxin in food, analogous p53 mutations in HCC were not found [87]. It was later demonstrated that the AFB1 mycotoxin acts as a cofactor during existing HBV infection, leading to enhanced hepatocarcinogenesis in these areas of the world [88].

#### C3. HCC and bacterial infections

Besides studies on the involvement of viruses and fungi, compelling data are also available on the role of bacteria in the development of HCC. An association between *H. pylori* and liver diseases has been established on the basis of numerous reports on identification of *H. pylori* DNA in various liver diseases [89-91].

Data from animal models suggest that bacterial microorganisms may directly induce carcinogenesis. Hepatic gene expression profiling in *H. hepaticus*-infected mice with advancing hepatocellular dysplasia revealed an up regulation of the putative tumor markers [92]. These tumor markers included H19, which activates insulin-like growth factor-II associated with hepatocarcinogenesis [93]. Moreover, acute-phase inflammatory proteins were also shown to be upregulated in the liver as a result of *H. hepaticus* infection [92].

Enteric *Helicobacter* bacteria also produce toxins that cause liver tissue damage [94]. It is well known that *H. hepaticus* produces a cytolethal distending toxin (CDT) with DNase activity [95], which may induce tumor development. As seen in CRC and GC, the VacA cytotoxin of *H. pylori* could also be involved in direct hepatocellular damage *in vivo *[12]*.*

#### C4. HCC and parasitic infections

Other than microorganisms, helminthes, all of which are trematodes, are strongly implicated in carcinogenesis [96]. The International Agency for Research on Cancer has categorized *Schistosoma haematobium* and *Opisthorchis viverrini* as group 1 and *Clonorchis sinensis* as group 2 carcinogens [97]. Infection with *S. haematobium* often leads to urinary bladder carcinoma, whereas the liver flukes *O. viverrini* and *C. sinensis* are linked to the development of bile duct cancer (cholangiocarcinoma) and liver cancer (hepatocarcinoma) in humans [98,99].

Although helminth-induced tumorigenesis may involve a number of complex mechanisms, chronic inflammation is a key feature [100]. Alternatively, parasite eggs and secreted products may cause physical damage leading to hyperplasia of the damaged liver tissue [90].

### D. Concluding remarks: Time for a change in the treatment paradigm?

Currently, approximately 15%–20% of all cancer cases worldwide and 26.3% of cancer cases in developing countries are attributable to pathogenic agents [96,101]. This equates to approximately 1,375,000 preventable cancer deaths per year [96]. It should not be surprising that one in every five malignant tumors can originate from pathogen-induced infection [102]. An increasing amount of data suggests that the frequency with which infectious agents contribute to cancer progression might be more than the aforementioned statistics. It is of crucial importance to consider the burden of infection caused by these pathogens due to the fact that both viral and microbial infections can accelerate the tumor development and tumor progression.

An understanding of the role that these pathogens play in tumor development may lead us towards new cancer treatment options that could subsequently increase the survival of cancer patients. This notion is supported by clinical data; it is reported that 83% of GCs were eradicated by treatment with the antibiotic, nitazoxanide [103]. To date, the 2-week antibiotic regimen for *H. pylori* has proved effective in reducing the prevalence of precancerous gastric lesions [104]. In addition to antibiotics, the frequent use of aspirin and non-steroidal anti-inflammatory drugs has been suggested as a protective factor in GC [105,106]. There was a 50% reduction in the incidence of GC in patients who took aspirin more often, i.e., 16 times a month, as well as a reduced gastric cancer risk in individuals taking non-aspirin non-steroidal anti-inflammatory drugs (NSAIDs). In EBVaGC, demethylating agents such as 5-aza cytidine are suggested as an effective treatment in lytic EBV infection [107].

For the treatment of CRC, thiazolides represent a novel class of antimicrobials against bacterial and viral pathogens, as well as helminthes and protozoan’s [108]. Thiazolides have been shown to induce glutathione S-transferase P1 (GSTP1)-dependent cell death in human colon cancer cells infected with a wide range of anaerobic bacteria, viruses, protozoan’s, and helminthes [109]. Their efficacy in CRC treatment can be attributed to their antimicrobial nature and this has been demonstrated by different laboratories [110,111]. Interestingly, it was also demonstrated that these antimicrobials induced apoptosis in CRC cells, with no significant side effects on normal colorectal cells [112].

Combined treatment with pegylated interferon plus ribavirin is now accepted as the standard therapy for HCC caused by chronic HCV infection [113]. HCV replication inhibitors, mainly HCV NS3/4A protease and NS5B polymerase, have been approved and are currently in phase II and III trials, which should significantly improve HCV treatment [114]. Similar antiviral strategies are used to treat virus-induced complications resulting in HCC development [115]. In addition to antiviral therapy, antimicrobial strategies are being successfully employed. The previously mentioned thiazolides have been shown to have a broad-spectrum activity against pathogens, not only in CRCs, but also in liver cancers and in other pathogen-induced GI carcinomas [110].

With accumulating data, it is becoming clear that there is a need to initiate adjuvant and prophylactic therapy long before starting traditional chemotherapy, especially in malignant GI tumors. This necessitates a detailed understanding of pathogens as promoters, initiators, or complicators of carcinogenesis. Comprehensive knowledge of pathogen-induced carcinogenesis in GI tract cancers will possibly help us reconsider our appreciation for antivirals and antimicrobials in cancer treatment and adapt an optimal approach to cancer therapy in the 21^st^ century.

## Abbreviations

GI: Gastrointestinal; *H. pylori*: *Helicobacter pylori*; CMV: Cytomegalovirus; EBV: Epstein-Barr virus; miRNAs: Micro RNAs; *H. felis*: *Helicobacter felis*; EBVaGC: EBV-associated gastric cancer; PTEN: Phosphatase and tensin homolog; cagA: Cytotoxin-associated gene A; vacA: Vacuolating cytotoxin gene A; GC: Gastric cancer; PAI: Pathogenicity island; BabA: Blood group antigen-binding adhesion; NF-?ß: Nuclear factor kappa beta; PGE2: Prostaglandin E2; C57BL/6: C57 black 6 mice strain; EBERs: Epstein-Barr virus-encoded small RNAs; LMP2A: Latent membrane protein 2A; NUGC- 3: Human gastric carcinoma line; p14ARF: Alternate reading frame product of the CDKN2A locus; p16Ink4A: Another name for cyclin-dependent kinase inhibitor 2A (CDKN2A); TIMP3: Tissue inhibitor of matrix metalloproteinases 3; DAPK: Death-associated protein kinase A; GSTP1: Glutathione S-transferase P1; JCV: JC virus or John Cunningham virus; pRb: Retinoblastoma protein; CRC: Colorectal cancer; *S. bovis*: *Streptococcus bovis*; IgG: Immunoglobulin G; ROS: Reactive oxygen species; HPV: Human papillomavirus; HCC: Hepatocellular carcinoma; HCV: Hepatitis C virus; HBV: Hepatitis B virus; NS3: NS4B, and NS5A, HCV non-structural proteins 3, 4B, and 5A, respectively; *H. hepaticus*: *Helicobacter hepaticus*; NSAID: Non-steroidal anti-inflammatory drug.

## Competing interests

Authors declare they have no competing interests.

## **Authors’ contributions**

KA, AD, AB, and BA performed the literature research and composed the manuscript. All authors read and approved the final manuscript.
